# 

**Published:** 1952-01

**Authors:** 


### *Contents*

| | | Page |
|---|---|---|
| EDITORIAL COMMENT | | 3 |
| ECZEMA OF THE COLON: A NEW CONCEPTION OF ULCERATIVE COLITIS | *Dr. John Naish* | 5 |
| MATERNITY SERVICE IN BRISTOL | *Professor G. Gordon Lennon* | 10 |
| THE MEDICAL WITNESS FOR THE PROSECUTION AND THE DEFENCE | *Dr. F. E. Camps* | 13 |
| POLIOMYELITIS IN BRISTOL, 1950 | *Dr. J. Macrae* | 20 |
| ANNOTATIONS | | 23 |
| REORGANIZATION OF THE AMBULANCE SERVICE | | |
| HOME NURSING EQUIPMENT | | |
| CLINICAL MEETINGS | | |
| BRISTOL MATERNITY FLYING SQUAD | | |
| CORRESPONDENCE: ROBBING PETER TO PAY PAUL | *Dr. Walter Woolley* | 25 |
| REPORTS OF MEETINGS | | 25 |
| BRISTOL MEDICO-CHIRURGICAL SOCIETY | | |
| DEVON AND EXETER MEDICO-CHIRURGICAL SOCIETY | | |
| SECOND INTERNATIONAL POLIOMYELITIS CONFERENCE | | |
| SOCIETY AND POST-GRADUATE PROGRAMMES | | 27 |
| APPOINTMENTS | | 30 |
| NOTES AND NEWS | | 31 |

